# Supplementary material for: Identification of motif-based interactions between SARS-CoV-2 protein domains and human peptide ligands pinpoint antiviral targets
Source: Nat Commun. 2023 Sep 13;14:5636. doi: 10.1038/s41467-023-41312-8 (PMC10499821; doi:10.1038/s41467-023-41312-8)
Supplement: Supplementary file 1 — Supplementary Information [file 41467_2023_41312_MOESM1_ESM.pdf]

Supplementary information

Identification of motif-based interactions between SARS-CoV-2 protein domains and human peptide ligands pinpoint antiviral targets

Filip Mihalič[1], Caroline Benz[2], Eszter Kassa[2], Richard Lindqvist[3,4], Leandro Simonetti[2], Raviteja Inturi[1], Hanna Aronsson[1], Eva Andersson[1], Celestine N. Chi[1], Norman E. Davey[5], Anna K. Överby[3,4], Per Jemth[1,*], and Ylva Ivarsson[2,*]

[1]Department of Medical Biochemistry and Microbiology, Uppsala University, Box 582, Husargatan 3, 751 23 Uppsala, Sweden

[2]Department of Chemistry - BMC, Uppsala University, Box 576, Husargatan 3, 751 23 Uppsala, Sweden

[3]Department of Clinical Microbiology, Umeå University, 90185 Umeå, Sweden

[4]Laboratory for Molecular Infection Medicine Sweden (MIMS), Umeå University, 90187 Umeå

[5]Division of Cancer Biology, The Institute of Cancer Research, 237 Fulham Road, London SW3 6JB, UK.

*Corresponding authors: Per.Jemth@imbim.uu.se; ylva.ivarsson@kemi.uu.se

Supplementary figures

Supplementary Figure 1. All affinity measurements performed in this study.

Supplementary Figure 2. Alignment of the peptides identified by ProP-PD screens.

Supplementary Figure 3. Fluorescence polarization experiments testing the importance of tyrosine 1084 in NCOA2$_{1074-1089}$ for binding to Nsp3 Ubl1.

Supplementary Figure 4. Models and confidence scores from ColabFold predictions for binding between Nsp3 globular domains and peptides identified in ProP-PD selections.

Supplementary Figure 5. SPOT array alanine scans for the PRDM14$_{197-213}$ peptide.

Supplementary Figure 6. Conservation of selected human peptides across animals.

Supplementary Figure 7. Model and confidence scores of ColabFold predictions for interaction between Nsp5 with DLG3$_{577-592}$ peptide.

Supplementary Figure 8. ColabFold for Nsp9

Supplementary Figure 9. Fluorescence polarization experiments of Nsp9 binding pocket mutant and the CD analysis of the Nsp9Δα

Supplementary Figure 10. Raw HSQC spectra and Nsp9-peptide model

Supplementary Figure 11. Surface representation of the Nsp9 NMR results

Supplementary Figure 12. ColabFold for Nsp16

Supplementary Figure 13. GST-pulldown experiments.

Supplementary Figure 14. ColabFold prediction of the peptide ligands identified in this study in the context of full-length proteins

Supplementary Figure 15. Cell penetrating peptides fail to inhibit HCoV 229E proliferation

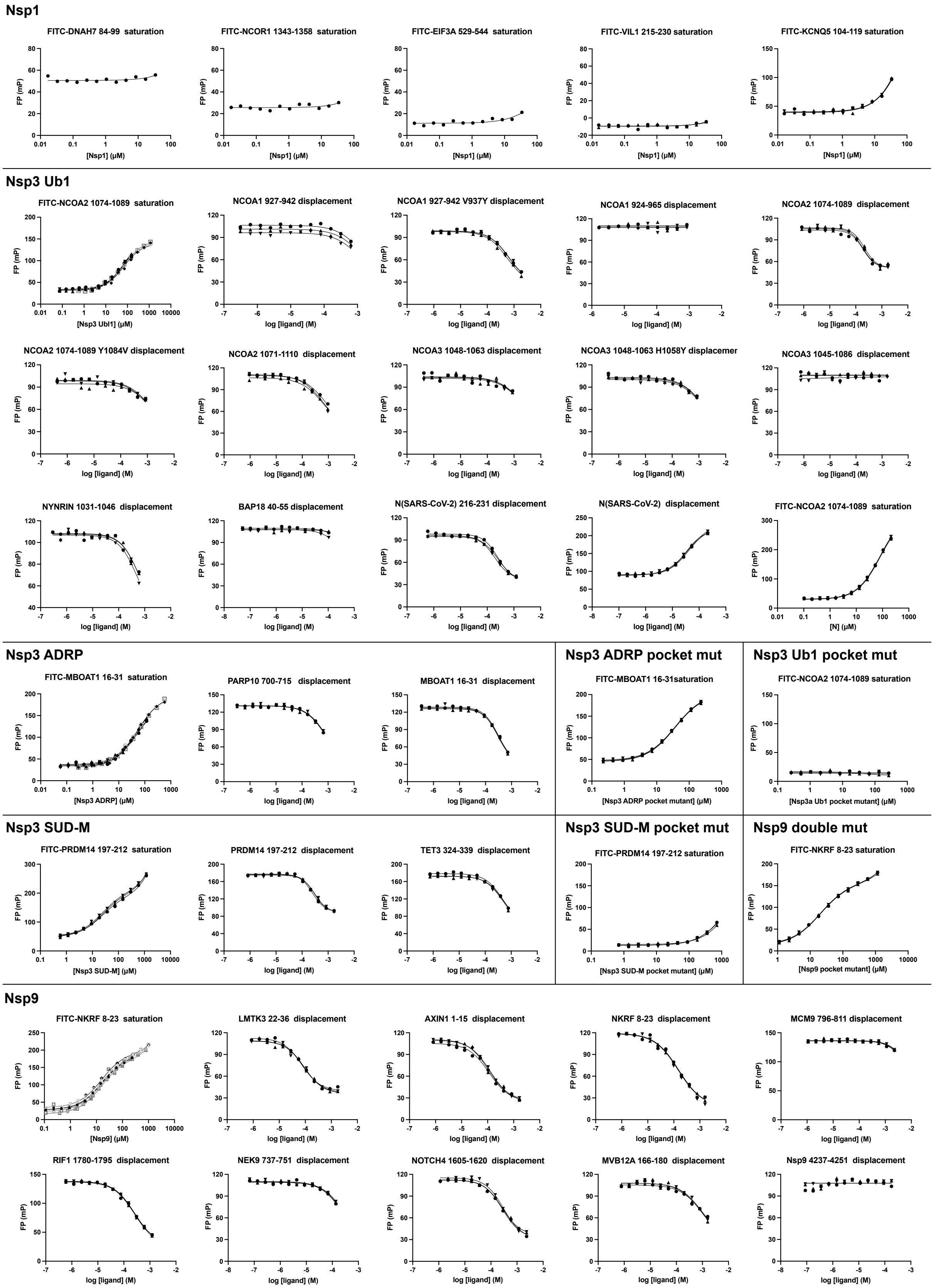
Nsp1
FITC-DNAH7 84-99 saturation
FITC-NCOR1 1343-1358 saturation
FITC-EIF3A 529-544 saturation
FITC-VIL1 215-230 saturation
FITC-KCNQ5 104-119 saturation
Nsp3 Ub1
FITC-NCOA2 1074-1089 saturation
NCOA1 927-942 displacement
NCOA1 927-942 V937Y displacement
NCOA1 924-965 displacement
NCOA2 1074-1089 displacement
NCOA2 1074-1089 Y1084V displacement
NCOA2 1071-1110 displacement
NCOA3 1048-1063 displacement
NCOA3 1048-1063 H1058Y displacemer
NCOA3 1045-1086 displacement
NYNRIN 1031-1046 displacement
BAP18 40-55 displacement
N(SARS-CoV-2) 216-231 displacement
N(SARS-CoV-2) displacement
FITC-NCOA2 1074-1089 saturation
Nsp3 ADRP
FITC-MBOAT1 16-31 saturation
PARP10 700-715 displacement
MBOAT1 16-31 displacement
Nsp3 ADRP pocket mut
FITC-MBOAT1 16-31saturation
Nsp3 Ub1 pocket mut
FITC-NCOA2 1074-1089 saturation
Nsp3 SUD-M
FITC-PRDM14 197-212 saturation
PRDM14 197-212 displacement
TET3 324-339 displacement
Nsp3 SUD-M pocket mut
FITC-PRDM14 197-212 saturation
Nsp9 double mut
FITC-NKRF 8-23 saturation
Nsp9
FITC-NKRF 8-23 saturation
LMTK3 22-36 displacement
AXIN1 1-15 displacement
NKRF 8-23 displacement
MCM9 796-811 displacement
RIF1 1780-1795 displacement
NEK9 737-751 displacement
NOTCH4 1605-1620 displacement
MVB12A 166-180 displacement
Nsp9 4237-4251 displacement
FP (mP)
[Nsp1] (μM)
[Nsp3 Ubl1] (μM)
log [ligand] (M)
[N] (μM)
[Nsp3 ADRP] (μM)
[Nsp3 ADRP pocket mutant] (μM)
[Nsp3a Ub1 pocket mutant] (μM)
[Nsp3 SUD-M] (μM)
[Nsp3 SUD-M pocket mutant] (μM)
[Nsp9 pocket mutant] (μM)
[Nsp9] (μM)

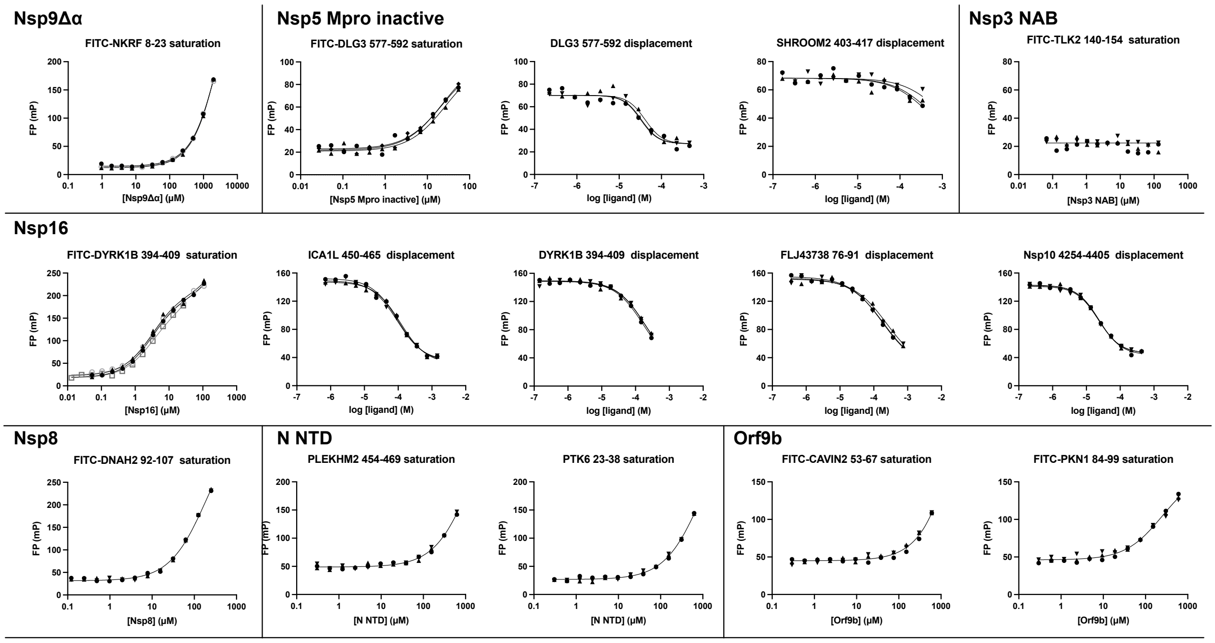

Supplementary Figure 1. All affinity measurements performed in this study. Peptide sequences and calculated K D values are presented in Supplementary Data 4. For all datasets the N^{3} 3 except for Nsp1. In the case of Nsp1, N=1 for FITC-DNAH784-99, FITC-NCOR11343-1358, FITC-EIF3A529-544, N=2 for FITC-VIL1215-230, and N=3 for KCNQ5104.

A

	Peptide sequence	Protein
**Nsp3 Ubl1**	631-QEDE**WV**NVQ**Y**PDQPEE-646	PLEKHM1
	973-NTFCTY**LL**EQ**I**D**M**LFF-988	PCNX2
	1031-CPSLSEE**IL**RC**L**S**L**HD-1046	NYNRIN
	286-NDDLVQR**LM**DI**LYA**SE-301	MAPRE2
	1074-SDEGA**LL**DQ**LYL**ALRN-1089	NCOA2
	1145-WSQVDEATIRA**II**AV**L**-1160	YTHDC2
	40-AKWTETEIEMLRAAVK-55	BAP18

B

	Peptide sequence	Protein
**Nsp3 ADRP**	18-GSTYL**HPLSE**LLGIPL-33	MBOAT1
	1-MAG**YLSE**SDFVMVEEG-16	AZIN2
	700-DGGTDGKAQLVVHSAF-715	PARP10

C

	Peptide sequence	Protein
**Nsp16**	452-DMSA**W**FN**LF**ADLDPLS-467	ICA1L
	447-KAASDLTA**W**FS**LF**ADL-462	ICA1
	394-PGHSPADYLR**F**QD**LV**L-409	DYRK1B
	78-PLDSYLN**F**QA**LI**SPRE-93	FLJ43738
	324-TDPLIRWDSYENLSAD-339	TNS3
	228-AHHTGSTASSEITPAQ-243	SCAPER

Supplementary Figure 2. Alignment of the peptides identified by ProP-PD screens. (A) Peptides identified by ProP-PD screens against Nsp3 Ubl1. Hydrophobic residues roughly corresponding to ΦxxΦxΦ are in bold. **(B)** Peptides identified by ProP-PD screens against Nsp3 ADRP. The common LSE motif is in bold. **(C)** Peptides identified by ProP-PD screens against Nsp16. The [W/F]xxxΦ motif is in bold.

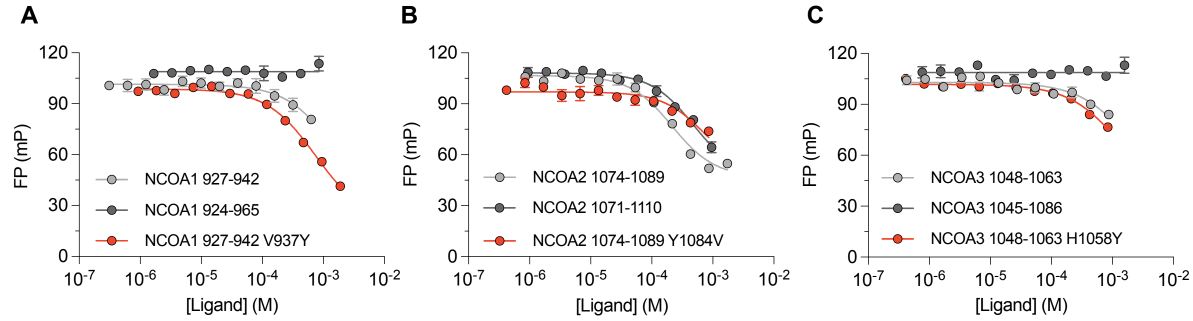

Supplementary Figure 3. Fluorescence polarization experiments testing the importance of tyrosine 1084 in NCOA2$_{1074\text{-}1089}$ for binding to Nsp3 Ubl1. (A) Comparison of short, long and V937Y mutant of NCOA1 peptide binder. **(B)** Comparison of short, long and Y1084V mutant of NCOA2 peptide binder. **(C)** Comparison of short, long and H1058Y mutant of NCOA3 peptide binder.

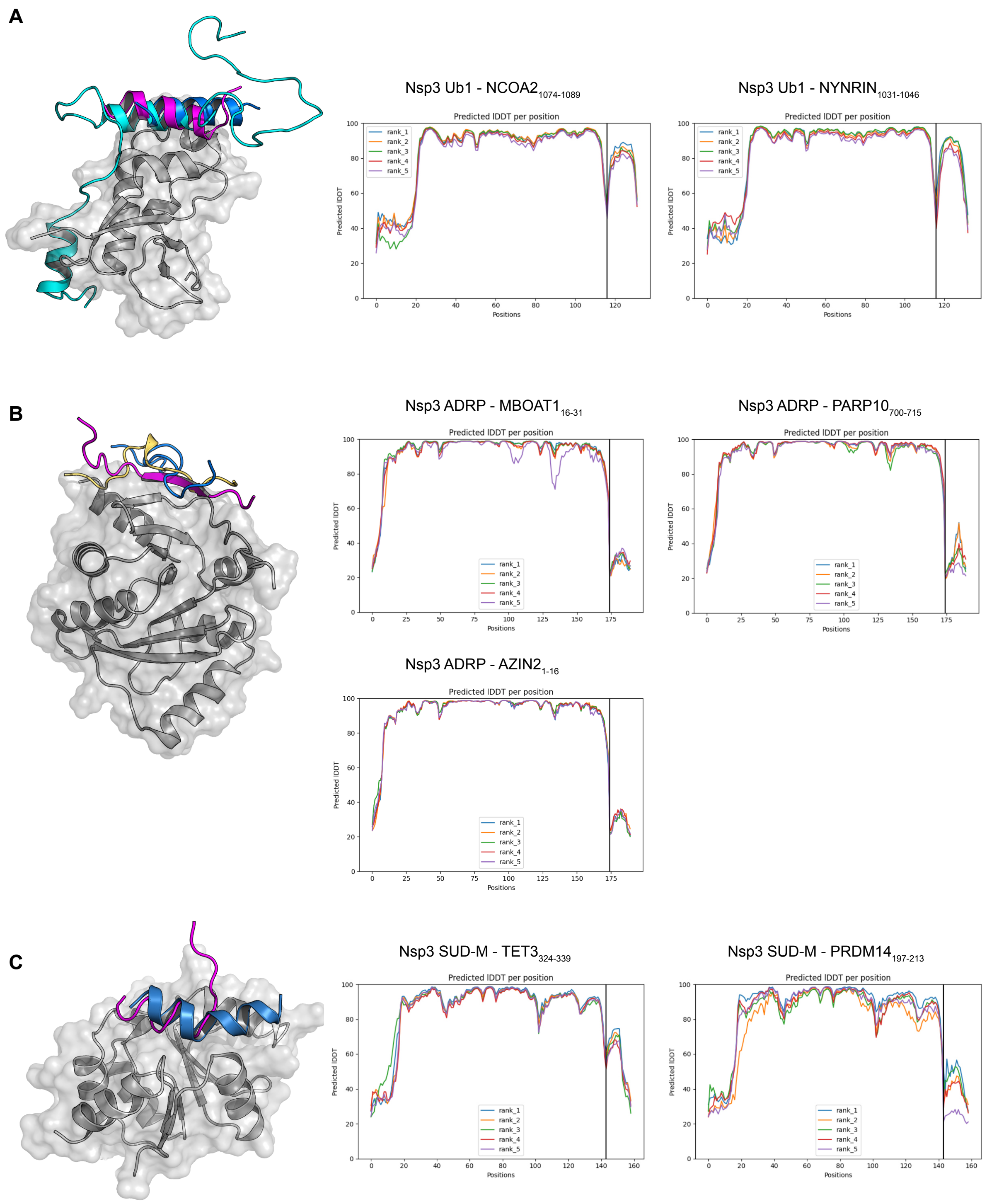

Supplementary Figure 4. Models and confidence scores from ColabFold predictions for binding between Nsp3 globular domains and peptides identified in ProP-PD selections. (A) Model and confidence scores for prediction of Nsp3 Ubl1 in complex with peptides. The vertical line in the lDDT graph of this and all subsequent lDDT graphs represent the break between the globular domain and the peptide. The NCOA2$_{1074\text{-}1089}$ peptide is in blue, and the NYNRIN$_{1031\text{-}1046}$ peptide is purple. In light blue the Ubl1 binding peptide from N (SARS-CoV-2) is superimposed (PDBid: 7PKU) **(B)** Model and confidence scores for prediction of Nsp3 ADRP in complex with

peptides. The $MBOAT1_{16-31}$ peptide is in blue, the $PARP10_{700-715}$ peptide is in purple, and the $AZIN_{1-16}$ peptide is yellow. **(C)** Model and confidence scores for prediction of Nsp3 SUD-M in complex with peptides. The $TET_{324-339}$ peptide is in blue, and the $PRDM14_{1074-1089}$ peptide is purple.

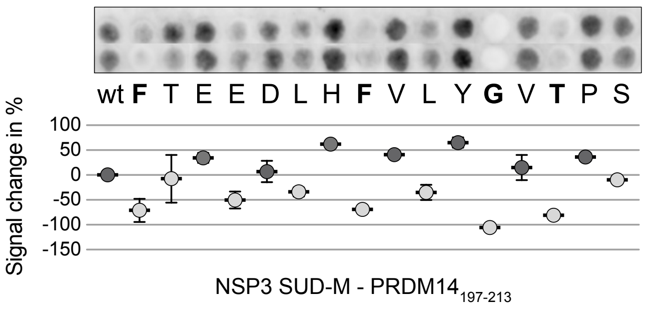

Supplementary Figure 5. SPOT array alanine scans for the PRDM14197-213 peptide. The peptide was challenged with Nsp3 SUD-M. The residues where clear reduction of binding is observed are in bold. Data are presented as mean ± SD (N = 2).

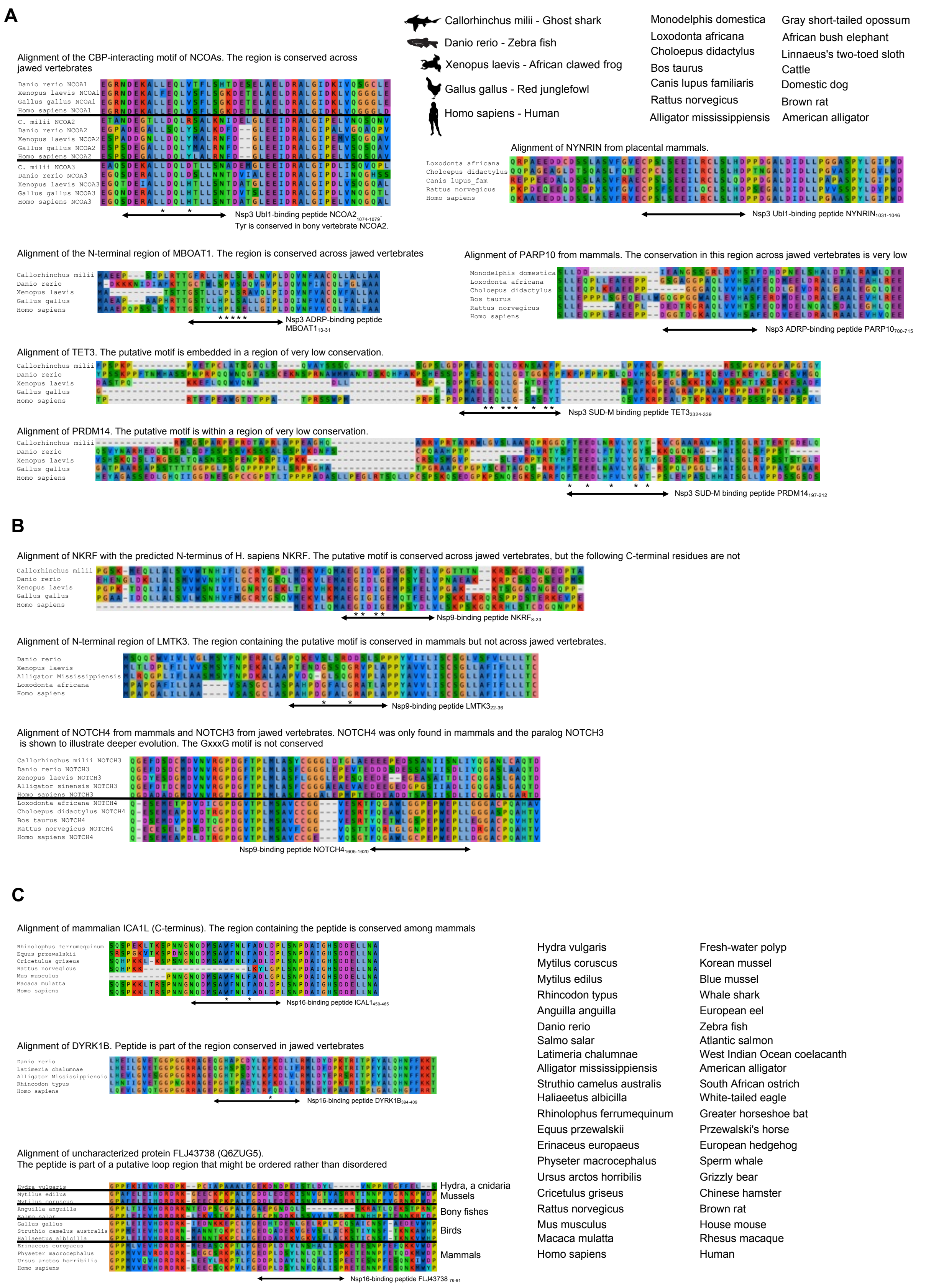

Supplementary Figure 6. Conservation of selected human peptides across animals. To investigate whether the peptides enriched in the ProP-PD selection against folded SARS-CoV-2 domains contain native binding motifs,

their sequence conservation between different animals was analyzed. The full-length human protein containing the peptide was aligned using the web version of Muscle[1] with orthologs or paralogs from selected animals in different branches of the animal kingdom. Only a small part of the alignment is shown, the region containing the peptide and its flanking sequences. The human 16- amino acid peptides that were enriched are indicated by arrows. Stars (*) denote that the amino acid position was confirmed by dot blot analysis to contribute to the binding of the SARS-CoV-2 protein domain. **(A)** Three domains from SARS-CoV-2 Nsp3 (Ubl1, ADRP and SUD-M) and the binding region for six human proteins, NCOA2, NYNRIN, MBOAT1, PARP10, TET3 and PRDM14. The entire region shown for NCOA2 (and its paralogs NCOA1 and 3) forms an interface with the NCBD domain in the transcriptional coregulator CBP/p300. **(B)** Three Nsp9-binding proteins: NKRF, LMTK3 and NOTCH4. The phylogeny among the paralogs in the NOTCH family is not clear. While NOTCH3 is found in all jawed vertebrates, NOTCH4 has so far only been found in mammals. **(C)** Three Nsp16-binding proteins: ICA1L, DYRK1B and FLJ43738 (unknown function). FLJ43738 is also called KIAA1257 and apparently present across the animal kingdom. The phylogeny with potential paralogs is not clear. See Supplementary Data 4 for K_D values and UniProt accession numbers.

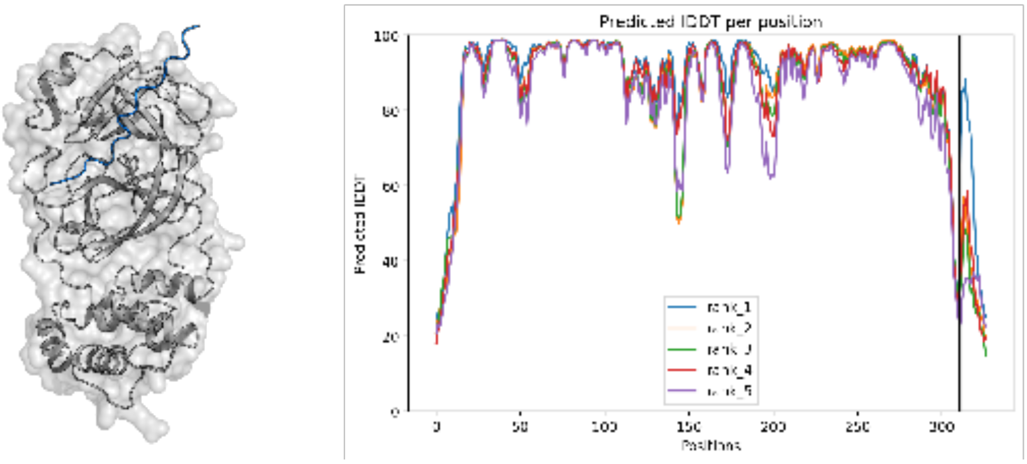

Supplementary Figure 7. Model and confidence scores of ColabFold predictions for interaction between Nsp5 with $DLG3_{577\text{-}592}$ peptide. Nsp5 is shown in gray cartoon, and peptide in blue ribbon. Only the rank one model is shown.

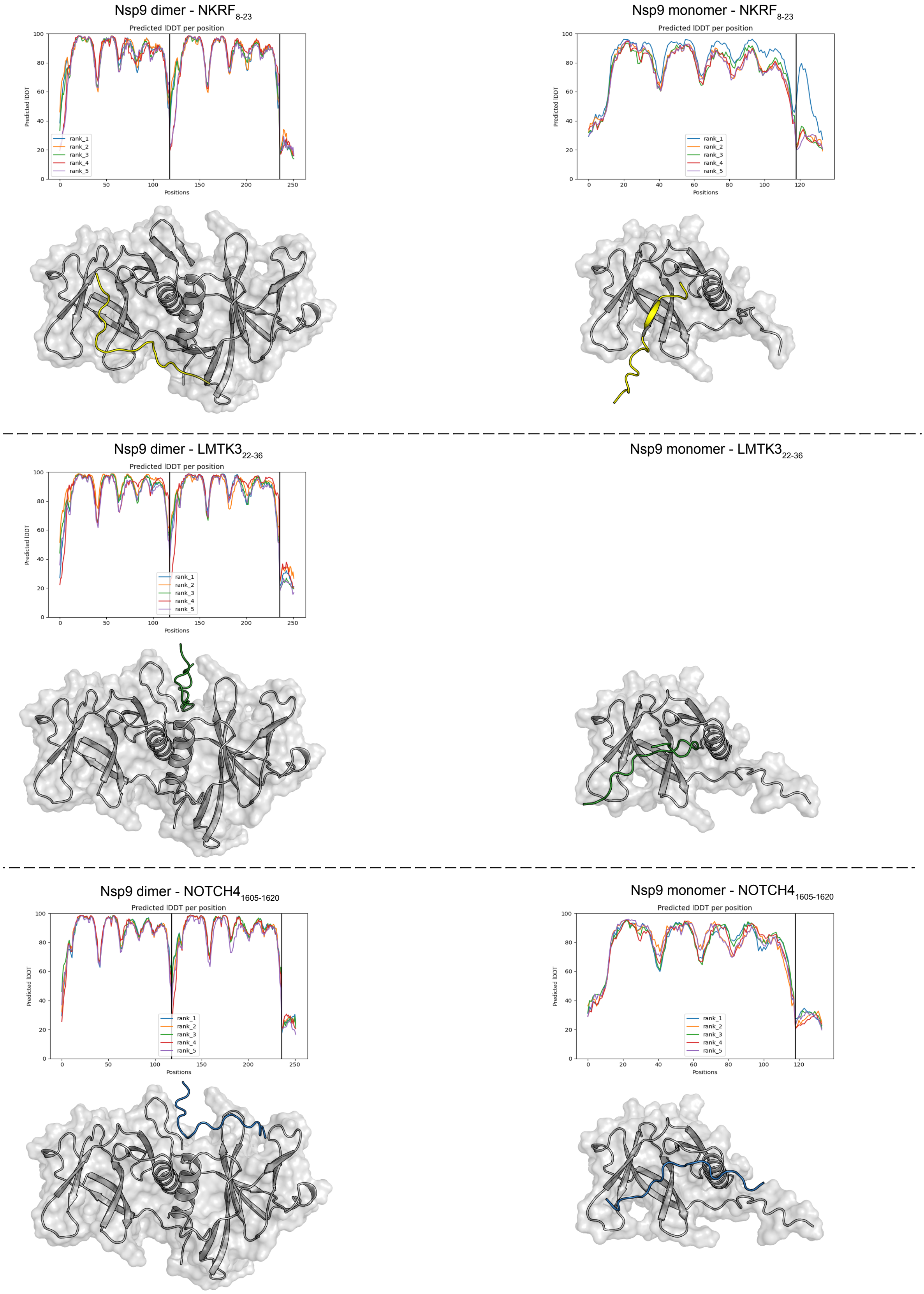

Supplementary Figure 8. Models and confidence scores of ColabFold predictions for interaction between Nsp9 with NKRF8-23, LMTK322-36, and NOTCH41605-1620 peptides. For all three peptides the prediciton was run with either monomeric or dimeric Nsp9. The predicted peptides did not converge with either Nsp9 monomer or dimer model.

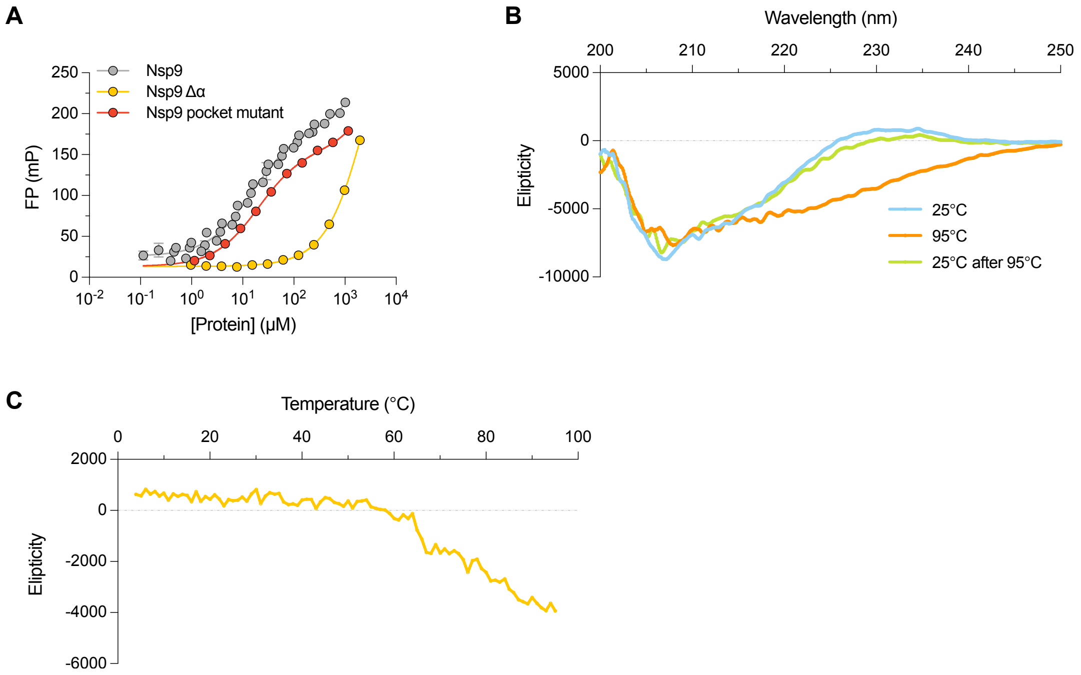

Supplementary Figure 9. Fluorescence polarization experiments of Nsp9 predicted binding pocket mutant and the CD analysis of the Nsp9Δα. (A) Comparison of the binding between FITC-NKRF$_{8-23}$ and Nsp9 (gray), Nsp9 with deleted C-terminal helix (Nsp9Δα; yellow) or Nsp9 binding pocket mutant (G4240N, G4244D; red). Data is represented as mean ± SEM (N = 3). (B) CD spectra of Nsp9Da before thermal denaturation (25°C), at 95°C and at 25°C after thermal denaturation. (N=4) (C) Thermal denaturation of the Nsp9Da construct moni tored by circular dichroism at 228 nm. Sigmoidal reduction in the CD signal indicates unfolding thus confirmi ng that at room temperature the construct is folded. (N=1)

A

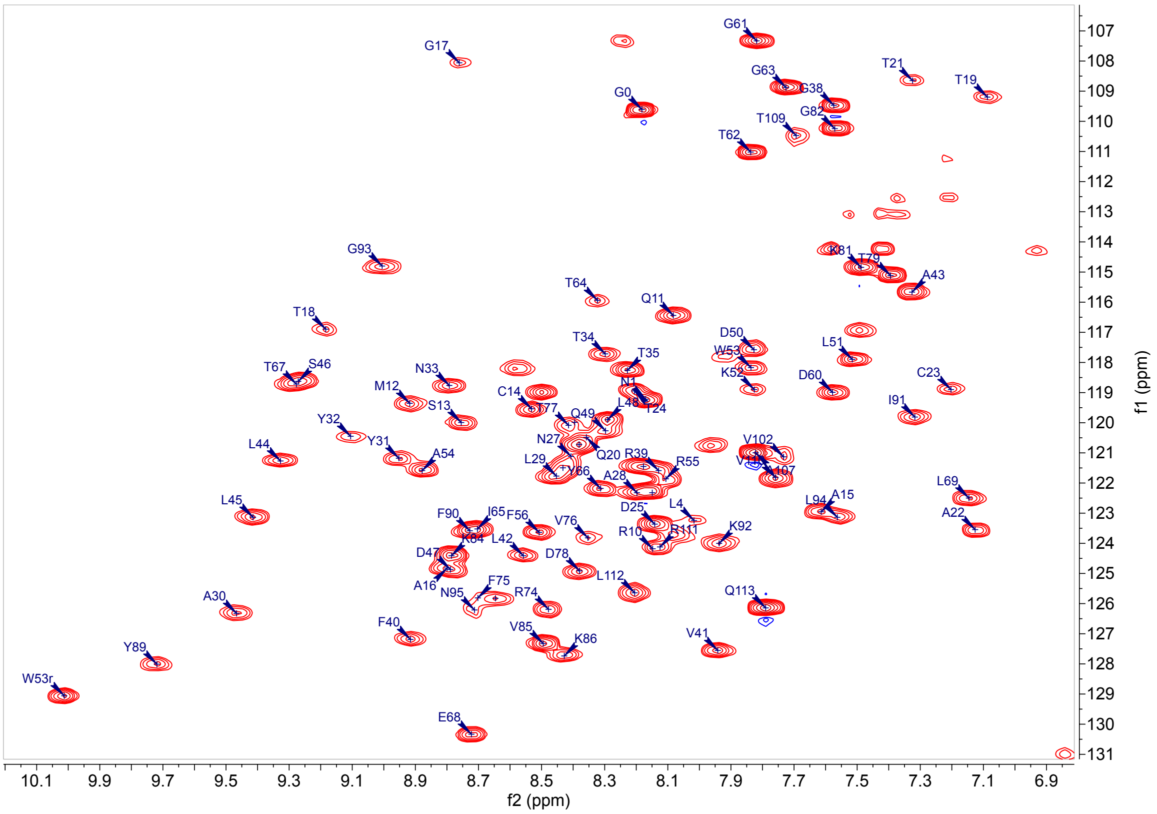

f2 (ppm)
f1 (ppm)
G61
G17
T21
G63
G38
T19
G0
T109
G82
T62
G93
K81
T79
A43
T64
Q11
T18
T34
T35
D50
L51
W53
T67
S46
N33
K52
D60
C23
M12
S13
C14
T77
Q49
L48
N1
T24
I91
Y32
Y31
N27
Q20
R39
V102
L44
A54
L29
Y66
R55
A107
A28
L69
L45
F90
I65
F56
V76
D25
L4
L94
A15
A22
K84
L42
R10
R111
K92
D47
D78
L112
A16
N95
F75
R74
Q113
A30
F40
V85
K86
V41
Y89
W53r
E68

B

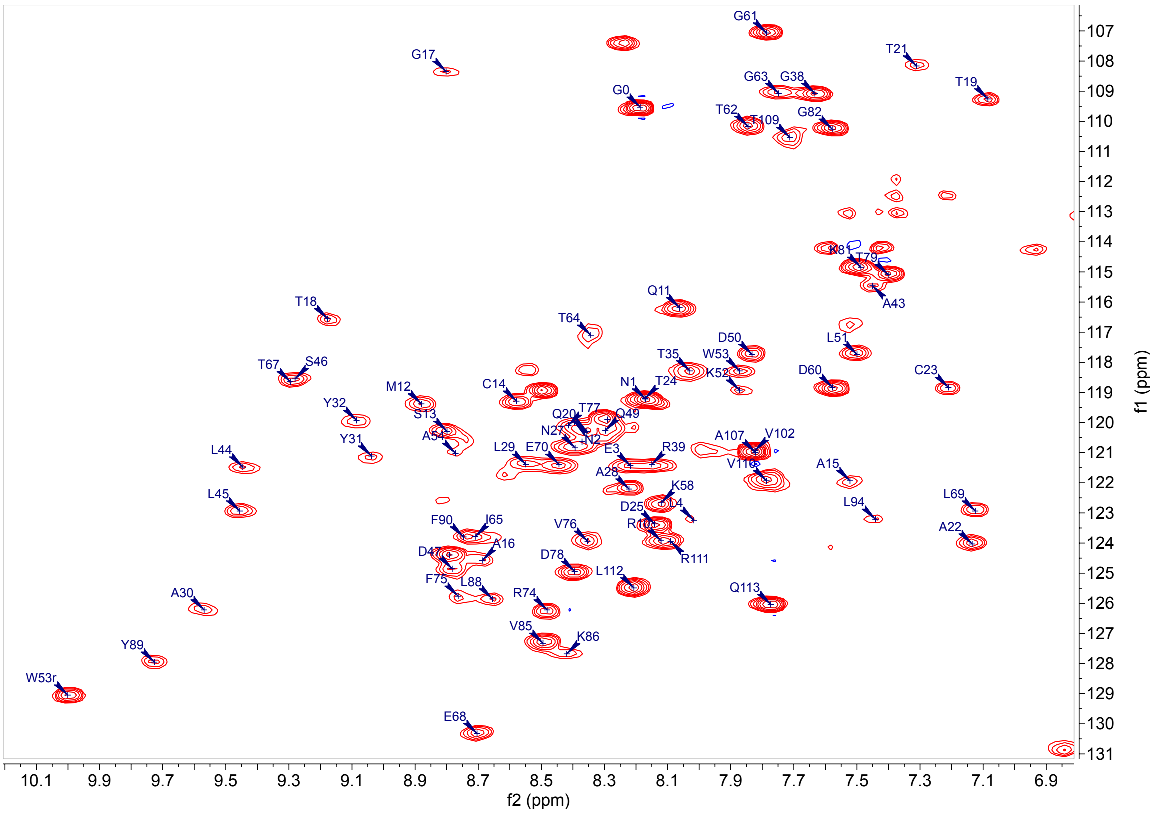

f2 (ppm)
f1 (ppm)
G61
G17
T21
G63
G38
T19
G0
T62
T109
G82
K81
T79
A43
Q11
T18
T64
D50
L51
T35
W53
K52
D60
C23
T67
S46
M12
C14
N1
T24
Y32
Q20
T77
Q49
S13
A54
N27
L29
E70
E3
R39
A107
V102
L44
A15
A28
K58
L69
L45
D25
L94
F90
I65
V76
A22
A16
D47
D78
L112
R111
F75
L88
R74
Q113
A30
V85
K86
Y89
W53r
E68

C

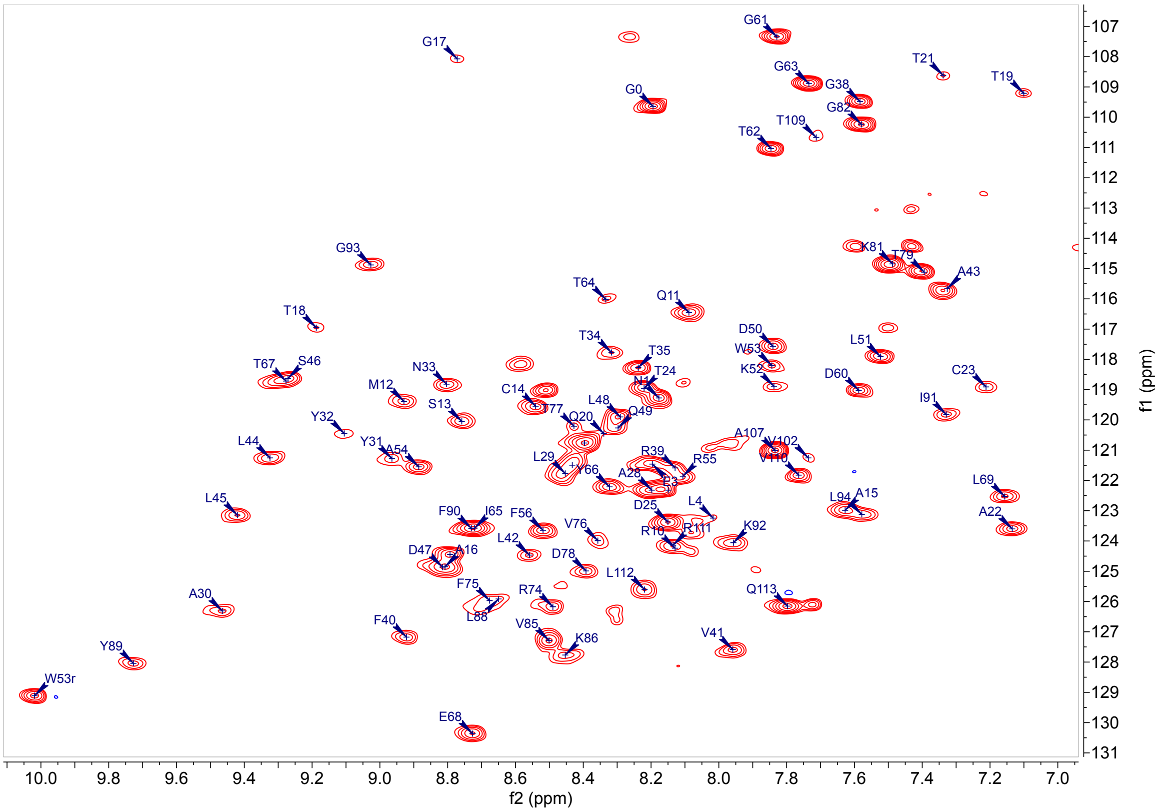

G61
G17
G63
T21
G38
T19
G0
G82
T62
T109
G93
K81
T79
A43
T64
Q11
T18
D50
T34
W53
L51
T35
T67
S46
K52
D60
C23
N33
T24
M12
N1
C14
L48
S13
I91
T77
Q49
Y32
Q20
A107
V102
Y31
A54
L44
V110
L29
R39
R55
Y66
A28
E3
L69
L45
L94
A15
D25
L4
F90
I65
F56
V76
R10
R111
K92
A22
L42
D47
A16
D78
L112
F75
R74
Q113
A30
L88
F40
V85
K86
V41
Y89
W53r
E68
f1 (ppm)
f2 (ppm)

D

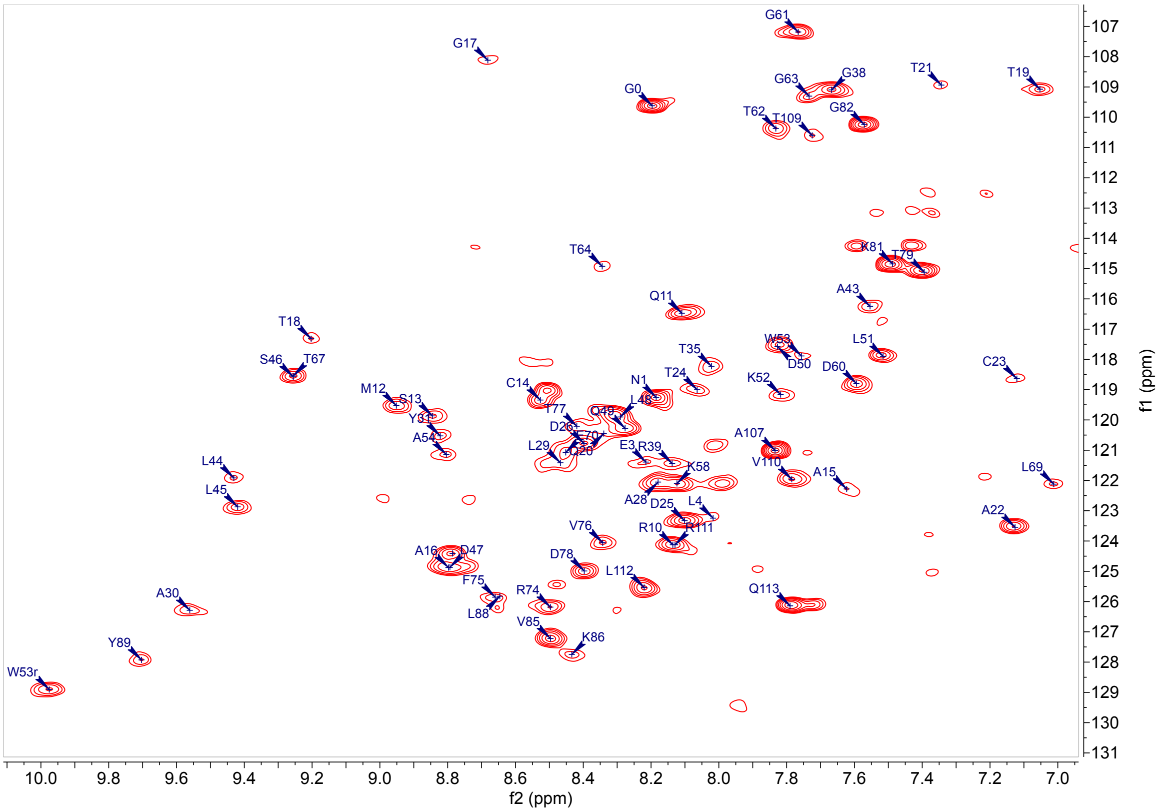

G61
G17
G63
G38
T21
T19
G0
T62
T109
G82
K81
T79
T64
A43
Q11
T18
L51
W53
D50
T35
S46
T67
K52
D60
C23
N1
T24
C14
M12
S13
L48
T77
Y31
Q49
A54
L29
Q20
E3
R39
K58
A107
V110
L44
A15
L69
L45
A28
D25
L4
A22
V76
R10
R111
A16
D47
D78
L112
F75
A30
R74
Q113
L88
V85
K86
Y89
W53r
f1 (ppm)
f2 (ppm)

E

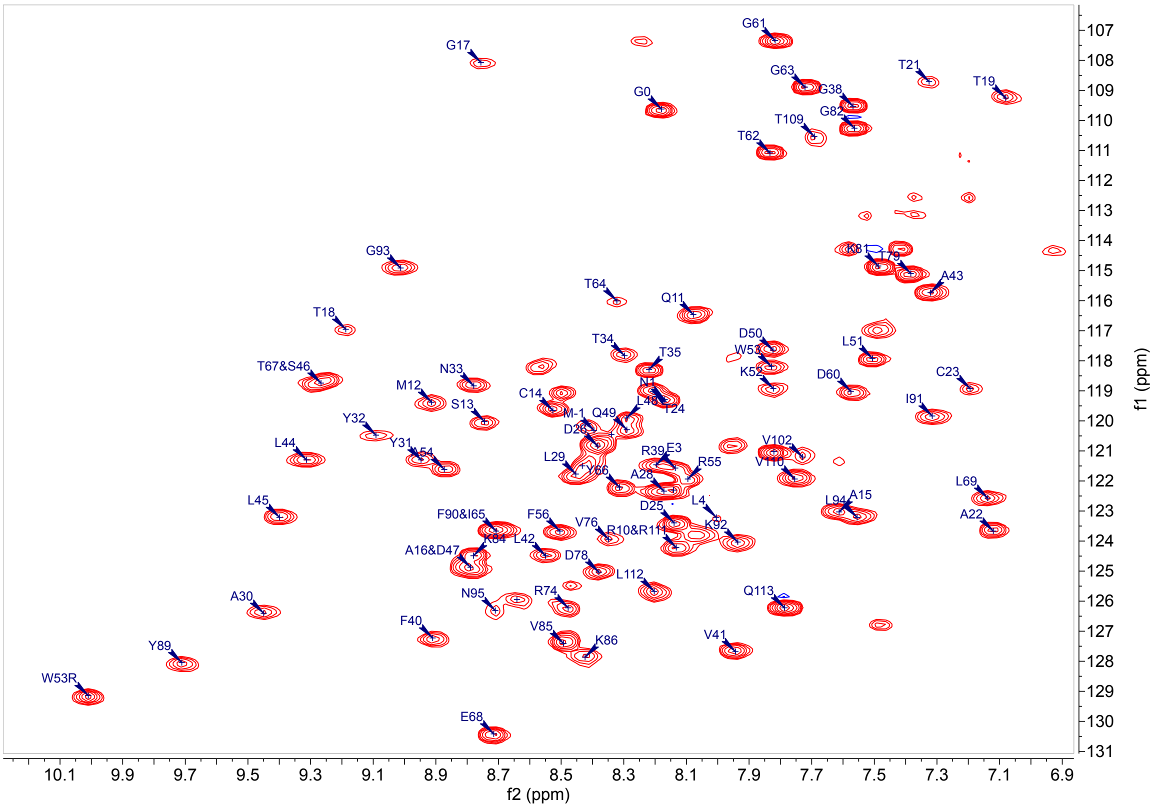
G61
G17
G63
T21
G38
T19
G0
T109
G82
T62
K81
T79
G93
A43
T64
Q11
T18
D50
T34
W53
L51
T35
T67&S46
N33
K52
D60
C23
M12
N1
L48
I91
C14
S13
T24
M-1
Q49
Y32
D26
V102
L44
Y31
A54
R39
E3
L29
V110
R55
Y66
L69
A28
L94
A15
L45
D25
L4
F90&I65
F56
V76
R10&R11
K92
A22
K84
L42
A16&D47
D78
L112
A30
N95
R74
Q113
F40
V85
K86
V41
Y89
W53R
E68
f2 (ppm)
f1 (ppm)

F

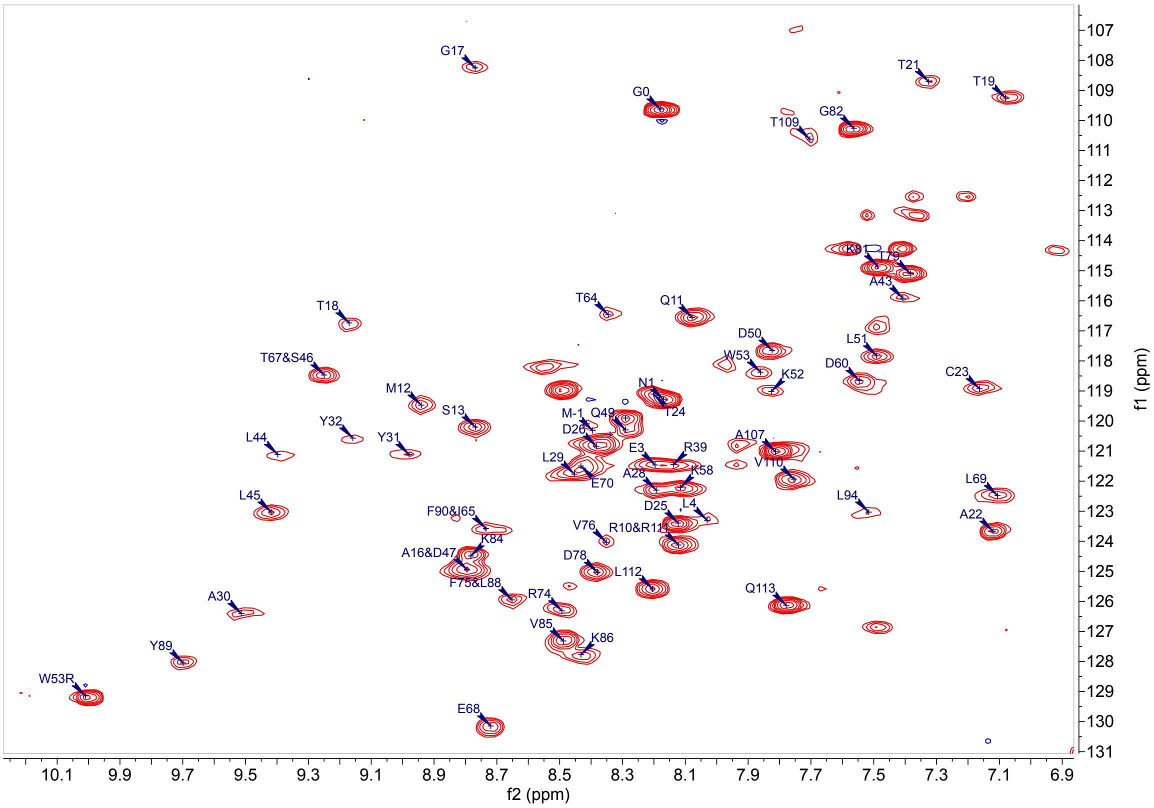
G17
T21
T19
G0
T109
G82
K81
T79
A43
T64
Q11
T18
D50
L51
T67&S46
W53
D60
K52
C23
M12
N1
S13
T24
M-1
Q49
Y32
D26
A107
L44
Y31
E3
R39
V110
L29
K58
A28
E70
L69
L45
L94
L4
D25
F90&I65
V76
R10&R11
A22
K84
A16&D47
D78
L112
F75&L88
A30
R74
Q113
V85
K86
Y89
W53R
E68
f2 (ppm)
f1 (ppm)

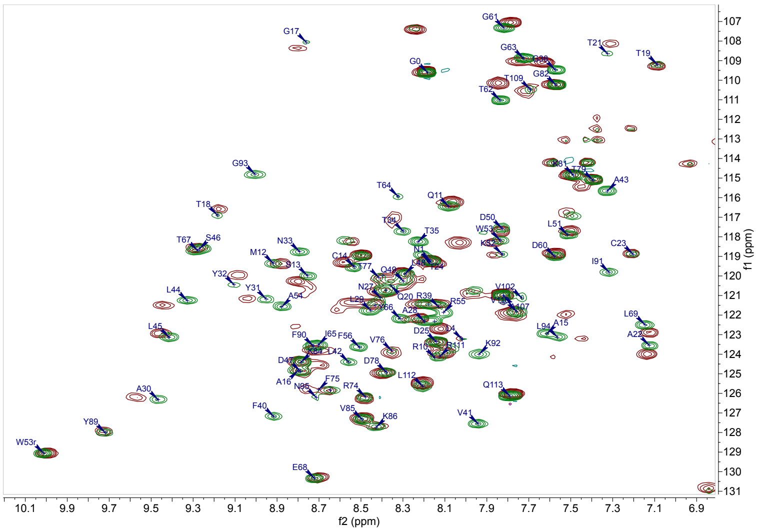

H

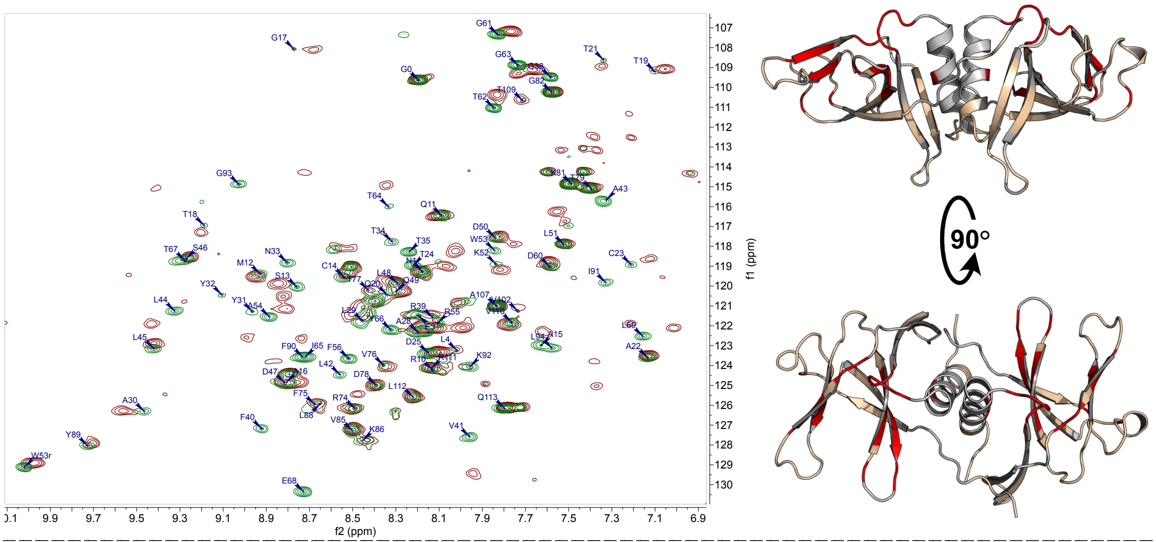

I

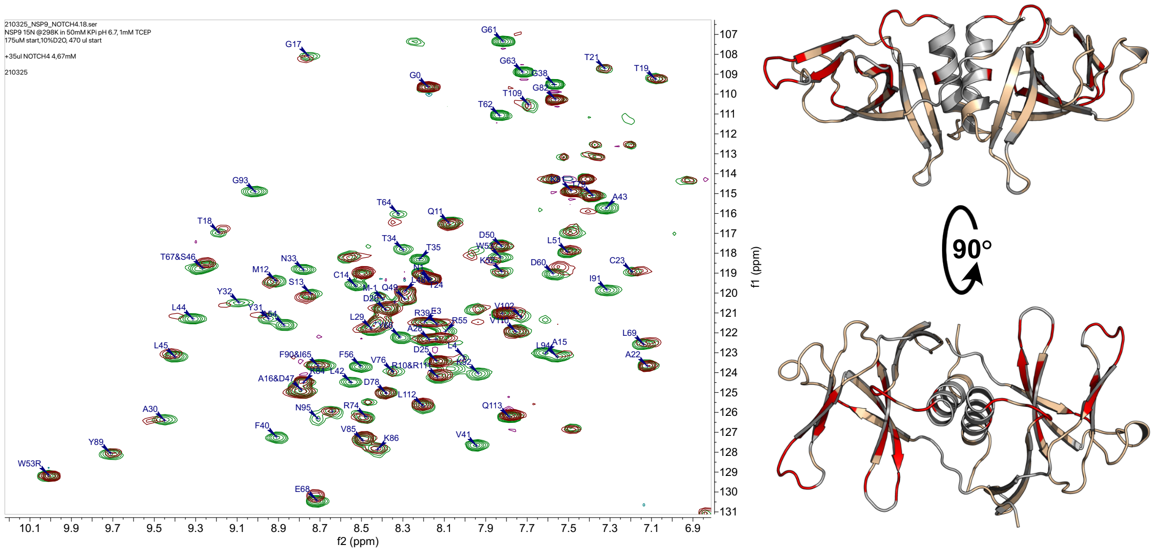

Supplementary Figure 10. Raw HSQC spectra and Nsp9 model with highlighted peak shift changes for NKRF$_{8\text{-}23}$, LMTK322-36 and NOTCH41605-1620 peptides.

(A) Chemical shift annotation for Nsp9 without added peptide for the experiment where NKRF8-23 was added

to the Nsp9. (B) Chemical shift annotation for Nsp9 with NKRF8-23 peptide.

(C) Chemical shift annotation for Nsp9 without peptide for the experiment where $LMTK3_{22-36}$ was added to the Nsp9. **(D)** Chemical shift annotation for Nsp9 with $LMTK3_{22-36}$ peptide. **(E)** Chemical shift annotation for Nsp9 without peptide for the experiment where $NOTCH4_{1605-1620}$ was added to the Nsp9. **(F)** Chemical shift annotation for Nsp9 with $NOTCH4_{1605-1620}$ peptide. **(G)** Overlay of spectra from **(A)** and **(B)**. **(H)** Overlay of spectra from **(C)** and **(D)**. On the right side the residues of Nsp9 with a chemical shift change larger than one standard deviation upon addition of $LMTK3_{22-36}$ are highlighted in red, the residues whose shift is bellow that threshold are in beige and the unassigned residues in gray. **(I)** Overlay of spectra from **(E)** and **(F)**. On the right side, residues of Nsp9 with a chemical shift change larger than one standard deviation upon addition of $NOTCH4_{1605-1620}$ are highlighted in red, the residues whose shift is bellow that threshold are in beige and the unassigned residues in gray.

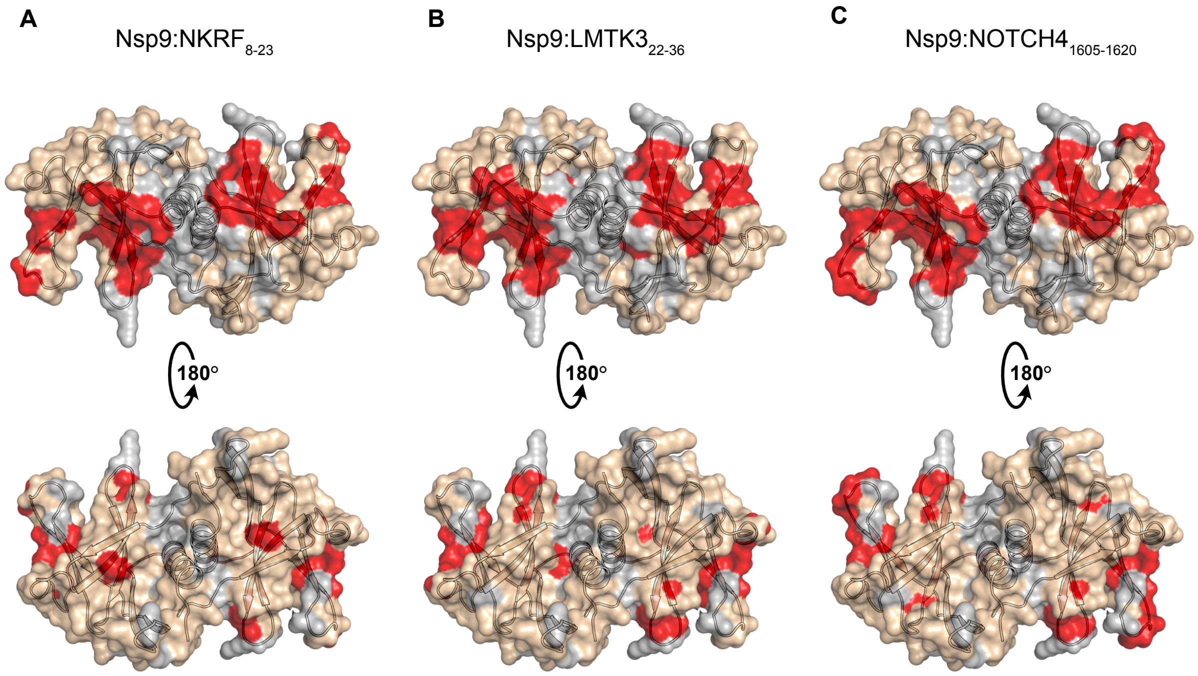

Supplementary Figure 11. Surface representation of the residues affected by the addition of peptide ligands as measured by NMR.

(A) Chemical shift perturbations upon addition of $NKRF_{8\text{-}23}$ peptide. The residues that were perturbed by more than one standard deviation are in red, the ones bellow that threshold are in beige and the residues that could not be assigned are in gray. Top view is the same as in the right panel of **Figure 5F**. **(B)** Chemical shift perturbations upon addition of peptide $LMTK3_{22\text{-}36}$. Coloring is the same is in (A). Top view is the same as in the bottom panel of **Figure S10H. (C)** Chemical shift perturbations upon addition of peptide $NOTCH4_{1605\text{-}1620}$. Coloring is the same is in (A). Top view is the same as in the bottom panel of **Figure S10I.**

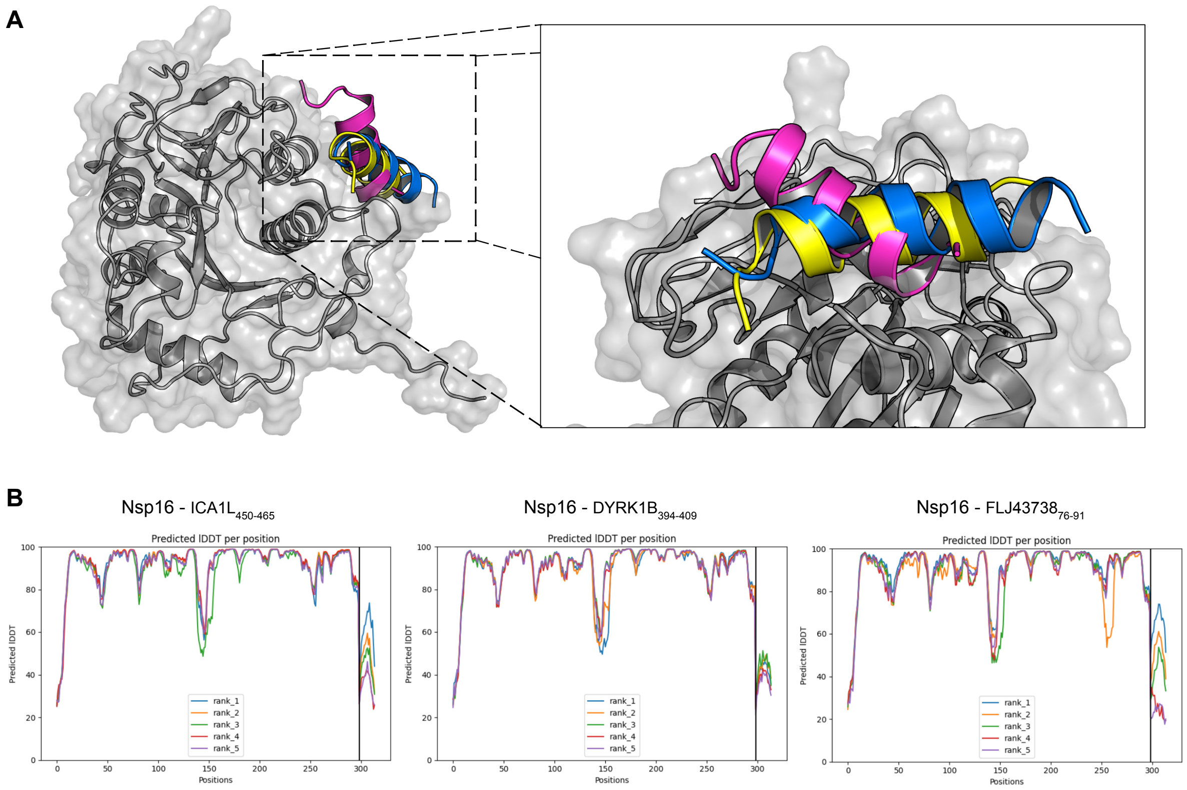

Supplementary Figure 12. Models and confidence scores of ColabFold predictions for the interaction between Nsp16 and DYRK1B395-410, ICA1L450-465, and FLJ4373876-91 peptides. (A) Superimposed best predicted model for the interaction. The ICA1L450-465 peptide is in blue the DYRK1B395-410 peptide in purple and the FLJ4373876-91 peptide is in yellow. (B) Confidence scores for the predictions.

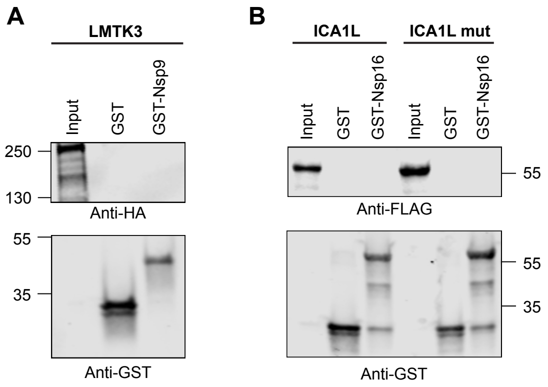

Supplementary Figure 13. GST-pulldown experiments. (A) Full length LMTK3 was expressed in HEK293T cells, and the capture was performed with GST-tagged Nsp9. (B) Full length ICA1L and ICA1L motif mutant (W456A, F460A) were expressed in HEK293T cells, and the capture was attempted with GST-tagged Nsp16. GST-tag alone serves as a negative control.

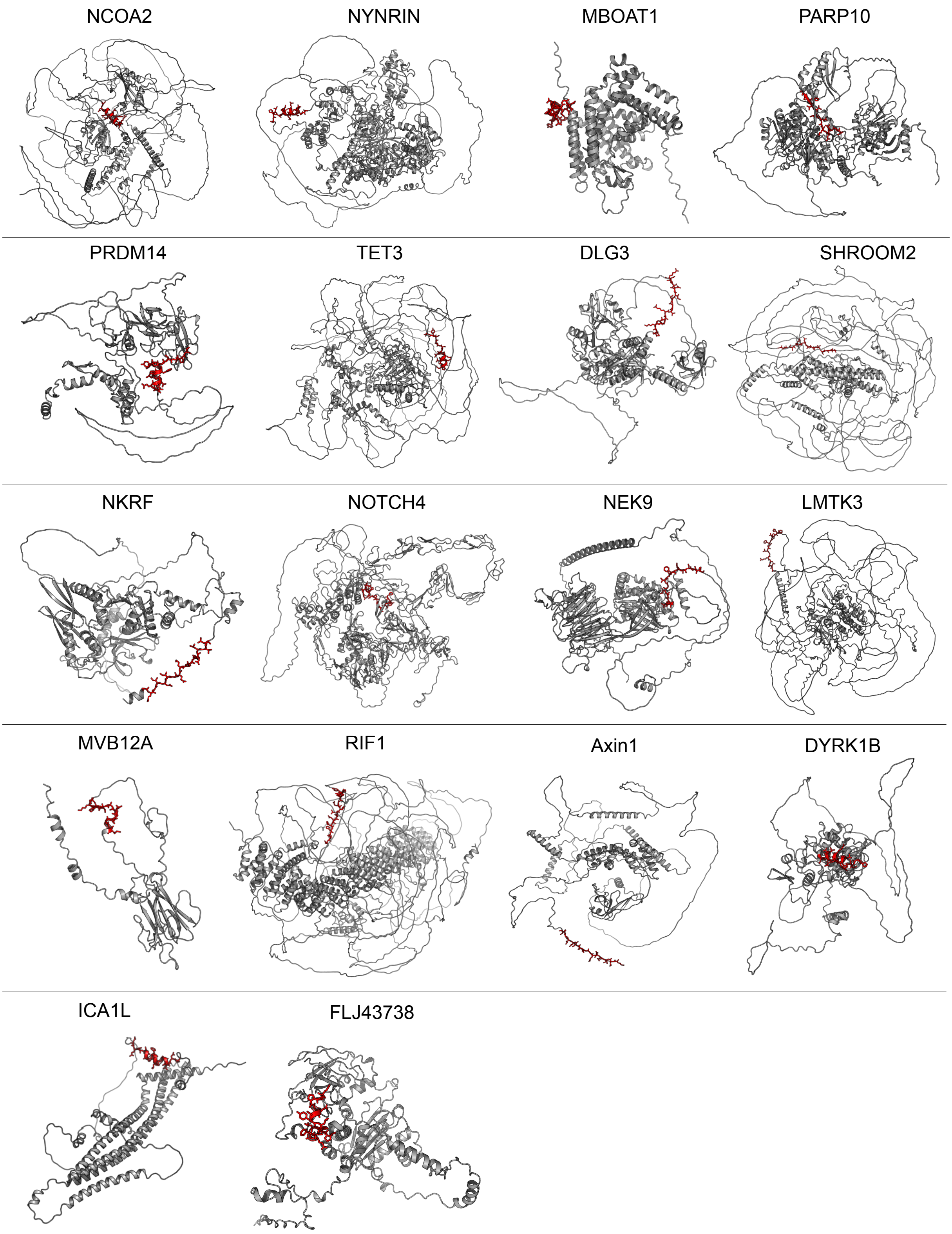

Supplementary Figure 14. ColabFold prediction of the peptide ligands identified in this study in the context of full-length proteins. The peptides in each case are shown as red sticks. In almost all cases the pepti des are part of the predicted disordered region.

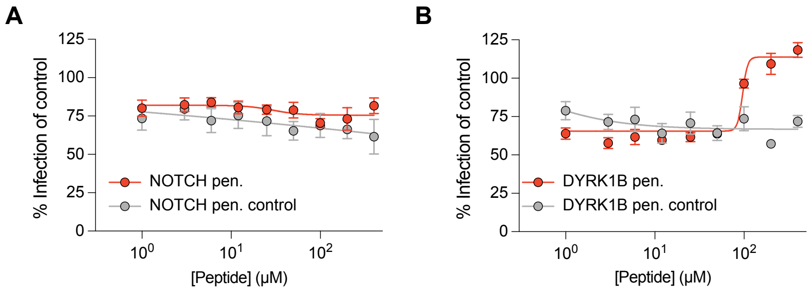

Supplementary Figure 15. Cell penetrating peptides fail to inhibit HCoV 229E proliferation. (A) The MRC5 cells were infected with HCoV 229E and subsequently treated with either NOTCH4 cell penetrating peptide (red dots) or the NOTCH4 control cell penetrating peptide (gray dots). (B) The same experiments were performed with the exception that the cells were treated with either DYRK1B cell penetrating peptide (red dots) or DYRK1B control cell penetrating peptide (gray dots).

Supplementary References

1 Edgar, R. C. MUSCLE: multiple sequence alignment with high accuracy and high throughput. *Nucleic Acids Res* **32**, 1792-1797 (2004). https://doi.org:10.1093/nar/gkh340
