# Supplementary material for: Identification of motif-based interactions between SARS-CoV-2 protein domains and human peptide ligands pinpoint antiviral targets
Source: Nat Commun. 2023 Sep 13;14:5636. doi: 10.1038/s41467-023-41312-8 (PMC10499821; doi:10.1038/s41467-023-41312-8)
Supplement: Supplementary file 3 — Description of Additional Supplementary Files [file 41467_2023_41312_MOESM3_ESM.pdf]

Description of Additional Supplementary Files

Supplementary Data 1. Overview of all SARS-CoV-2 protein domain constructs used in this study

Supplementary Data 2. ProP-PD selection results

Supplementary Data 3. GO term analysis

Supplementary Data 4. Overview of the peptides and affinity measurements

Supplementary Data 5. NMR peak shift perturbation calculation

Supplementary Data 6. NMR *T1, T2* calculation

Supplementary Data 7. Lentiviral constructs used in this study

Supplementary Data 8. Cell penetrating peptides
