# Supplementary material for: Identification of motif-based interactions between SARS-CoV-2 protein domains and human peptide ligands pinpoint antiviral targets
Source: Nat Commun. 2023 Sep 13;14:5636. doi: 10.1038/s41467-023-41312-8 (PMC10499821; doi:10.1038/s41467-023-41312-8)
Supplement: Supplementary file 12 — Reporting Summary [file 41467_2023_41312_MOESM12_ESM.pdf]

nature portfolio

Corresponding author(s): Per Jemth

Last updated by author(s): Aug 15, 2023

Reporting Summary

Nature Portfolio wishes to improve the reproducibility of the work that we publish. This form provides structure for consistency and transparency in reporting. For further information on Nature Portfolio policies, see our Editorial Policies and the Editorial Policy Checklist.

Statistics

For all statistical analyses, confirm that the following items are present in the figure legend, table legend, main text, or Methods section.

n/a	Confirmed	
☐	☒	The exact sample size (*n*) for each experimental group/condition, given as a discrete number and unit of measurement
☐	☒	A statement on whether measurements were taken from distinct samples or whether the same sample was measured repeatedly
☐	☒	The statistical test(s) used AND whether they are one- or two-sided<br>*Only common tests should be described solely by name; describe more complex techniques in the Methods section.*
☒	☐	A description of all covariates tested
☒	☐	A description of any assumptions or corrections, such as tests of normality and adjustment for multiple comparisons
☐	☒	A full description of the statistical parameters including central tendency (e.g. means) or other basic estimates (e.g. regression coefficient) AND variation (e.g. standard deviation) or associated estimates of uncertainty (e.g. confidence intervals)
☐	☒	For null hypothesis testing, the test statistic (e.g. *F*, *t*, *r*) with confidence intervals, effect sizes, degrees of freedom and *P* value noted<br>*Give P values as exact values whenever suitable.*
☒	☐	For Bayesian analysis, information on the choice of priors and Markov chain Monte Carlo settings
☒	☐	For hierarchical and complex designs, identification of the appropriate level for tests and full reporting of outcomes
☒	☐	Estimates of effect sizes (e.g. Cohen's *d*, Pearson's *r*), indicating how they were calculated

Our web collection on statistics for biologists contains articles on many of the points above.

Software and code

Policy information about availability of computer code

Data collection	For alanine scanning SPOT arrays: ChemiDoc Imaging system (Bio-Rad),<br>For the acquisition of NMR spectra:TopSpin 3.2,<br>For Circular dichroism: Spectra Manager Version 2.13<br>For western blots: Image Studio Version 5.2<br>For cell penetrating peptide analysis: Leica Application Suit X software (LAS X, Leica)<br>For the acquisition of fluorescence polarization results: SoftMax Pro 7.0.3
Data analysis	Image Studio Lite Ver. 5.2 (LI-COR)<br>For the analysis of NMR experiments: MestReNova 14.1.0.,<br>For the analysis of fluorescence polarization and viral infection assay experiments: GraphPad Prism 9 (version 9.4.1),<br>to facilitate the visualization of Colabfold predictions and NMR experimental results: PyMOL 2.5.4

For manuscripts utilizing custom algorithms or software that are central to the research but not yet described in published literature, software must be made available to editors and reviewers. We strongly encourage code deposition in a community repository (e.g. GitHub). See the Nature Portfolio guidelines for submitting code & software for further information.

Data

Policy information about availability of data

All manuscripts must include a data availability statement. This statement should provide the following information, where applicable:

- Accession codes, unique identifiers, or web links for publicly available datasets
- A description of any restrictions on data availability
- For clinical datasets or third party data, please ensure that the statement adheres to our policy

The details of the library designs including the proteins, peptides and statistics are available at http://slim.icr.ac.uk/phage_libraries/human/, and were described previously (PMID: 35044719). The PDB structures from PDBid: 6wxd [http://doi.org/10.2210/pdb6WXD/pdb] and 7jyy [http://doi.org/10.2210/pdb7JYY/pdb] were used in this study. Source data for this study are provided with this paper.

Human research participants

Policy information about studies involving human research participants and Sex and Gender in Research.

Reporting on sex and gender	n/a
Population characteristics	n/a
Recruitment	n/a
Ethics oversight	n/a

Note that full information on the approval of the study protocol must also be provided in the manuscript.

Field-specific reporting

Please select the one below that is the best fit for your research. If you are not sure, read the appropriate sections before making your selection.

☒ Life sciences ☐ Behavioural & social sciences ☐ Ecological, evolutionary & environmental sciences

For a reference copy of the document with all sections, see nature.com/documents/nr-reporting-summary-flat.pdf

Life sciences study design

All studies must disclose on these points even when the disclosure is negative.

Sample size	No sample-size was performed using statistical methods. The sample sizes were determined based on similar previous work (https://doi.org:10.1038/s41467-021-26498-z; https://doi.org:10.1101/2022.06.19.496705) and expected effect sizes observed for the types of assays employed in this study.
Data exclusions	No predetermined exclusion criteria were established.
Replication	Our experimental findings were reliably reproduced through repeated experiments and using different approaches. The number of biologically independent replicates and individual data points are indicated in the figure legends.
Randomization	The experiments were not randomized as the randomization was judged not possible or needed for the types of experiments performed in this study, as work was performed with purified proteins and peptides, and not live subjects. Additionally, control experiments were performed to reduce bias.<br>For phage display selections: protein baits were randomly distributed in 96 well plates.
Blinding	The blinding was not relevant for the types of experiments performed in this study because the effects measured in this study were well defined and control experiments were employed to prevent biased results.

Reporting for specific materials, systems and methods

We require information from authors about some types of materials, experimental systems and methods used in many studies. Here, indicate whether each material, system or method listed is relevant to your study. If you are not sure if a list item applies to your research, read the appropriate section before selecting a response.

Materials & experimental systems

n/a	Involved in the study
☐	☒ Antibodies
☐	☒ Eukaryotic cell lines
☒	☐ Palaeontology and archaeology
☒	☐ Animals and other organisms
☒	☐ Clinical data
☒	☐ Dual use research of concern

Methods

n/a	Involved in the study
☒	☐ ChIP-seq
☒	☐ Flow cytometry
☒	☐ MRI-based neuroimaging

Antibodies

Antibodies used

1) HRP-conjugated M13 bacteriophage antibody (Sino Biological; 11973-MM05T-H; Monoclonal Mouse IgG1 Clone #MM05; 1:5,000 dilution,
2) HRP-conjugated anti-GST antibody (Cytiva, RPN1236; 1:3000 dilution),
3) Primary monoclonal rabbit antibodies directed against SARS CoV-2 nucleocapsid (Sino Biological Inc., 40143-R001; 1:500 dilution),
4) Primary monoclonal mouse antibodies J2 directed against dsRNA (Scicons 10010500; 1:500 dilution)
5) Primary monoclonal mouse anti-Flag (Sigma, M2, F1804; 1:1000 dilution)
6) Primary monoclonal mouse anti-HA (Biolegend, 901501; 1:1000 dilution)
7) Primary polyclonal goat anti-GST (Pharmacia, 27-4577; 1:1000 dilution)
8) Secondary polyclonal goat anti-mouse IgG (LI-COR, 926-32210; 1:10000 dilution)
9) Secondary polyclonal donkey anti-goat IgG (LI-COR, 926-68074; 1:10000 dilution)
10) Secondary donkey anti-Rabbit IgG (H+L) Highly Cross-Adsorbed Secondary Antibody, Alexa Fluor 555, (Invitrogen, A31572; 1:500 dilution)
11) Secondary donkey anti-Mouse IgG (H+L) Highly Cross-Adsorbed Secondary Antibody, Alexa Fluor 555, (Invitrogen, A31570; 1:500 dilution)

Validation

All antibodies have been validated by the manufacturer and in other publications. For immunofluorescence the presence of signal in the cells expressing viral proteins or viral RNA or the lack of signal in the absence of infection was used as validation.

1) HRP-conjugated M13 bacteriophage antibody (Sino Biological; 11973-MM05T-H). Validated applications: ELISA (https://www.sinobiological.com/antibodies/m13-11973-mm05t-h). Previouse publications: Provided at the producers web page. (Khalil, A.S. et al., 2007, Proc Natl Acad Sci. USA. 104 (12): 4892-7)

2) Anti-GST-HRP conjugate: Horseradish Peroxidase (HRP) conjugated to goat anti-GST polyclonal antibody (Cytiva, RPN1236). (https://www.cytivalifesciences.com/en/us/shop/protein-analysis/blotting-and-detection/blotting-standards-and-reagents/anti-gst-hrp-conjugate-p-05747#overview)

3) SARS-CoV/SARS-CoV-2 Nucleocapsid Antibody, Rabbit MAb (Sino Biological Inc., 40143-R001). Validated applications: WB, ELISA, IHC-P, ICC/IF (https://www.sinobiological.com/antibodies/cov-nucleocapsid-40143-r001). Previous publications: PMID: 34799561, 32847628.

4) anti-dsRNA mAb J2 (Scicons 10010500). Validated applicaitons: Immunofluorescence, immunehistology, immune electronmicroscopy, immunoblotting, immunprecipitation, ELISA (https://www.labome.com/product/SCICONS/10010500.html). Previous publications: PMID:29411137, 29370303.

5) anti-Flag (Sigma, M2, F1804). Validated applicaitons: For highly sensitive and specific detection of FLAG fusion proteins by immunoblotting, immunoprecipitation (IP), immunohistochemisty, immunofluorescence and immunocyotchemistry. Optimized for single banded detection of FLAG fusion proteins in mammalian, plant, and bacterial expression systems. (https://www.sigmaaldrich.com/SE/en/product/sigma/f1804). Previous publications: PMID: 37100772 (and many others)

6) mouse anti-HA (Biolegend, 901501). Validated applicaitons: WB - Quality tested, FC, ICC, IP - Verified, Purification - Reported in the literature, not verified in house (https://www.biolegend.com/en-us/products/purified-anti-ha-11-epitope-tag-antibody-11374). Previous publications: Provided at the producers web page.

7) goat anti-GST (Pharmacia, 27-4577). Application:Western Blots, (https://www.sigmaaldrich.com/SE/en/product/sigma/ge27457701). Previous publications: PMID: 37225695, (and many others)

8) goat anti-mouse IgG (LI-COR, 926-32210). Application:Western Blots, (https://www.licor.com/bio/reagents/irdye-800cw-goat-anti-mouse-igg-secondary-antibody)

9) donkey anti-goat IgG (LI-COR, 926-68074). Application:Western Blots, (https://www.licor.com/bio/reagents/irdye-680rd-donkey-anti-goat-igg-secondary-antibody)

10) donkey anti-Rabbit IgG (H+L) Highly Cross-Adsorbed Secondary Antibody, Alexa Fluor 555, (Invitrogen, A31572). Application:Western Blots, Immunohistochemistry (IHC) and others (see: https://www.thermofisher.com/antibody/product/Donkey-anti-Rabbit-IgG-H-L-Highly-Cross-Adsorbed-Secondary-Antibody-Polyclonal/A-31572). References to the papers which used same antibody can be found at producers web page (example: Cariulo C, Martufi P, Verani M, Toledo-Sherman L, Lee R, Dominguez C, Petricca L, Caricasole A. IKBKB reduces huntingtin aggregation by phosphorylating serine 13 via a non-canonical IKK pathway. Life Sci

Alliance. 2023 Aug 8;6(10):e202302006. doi: 10.26508/lsa.202302006. PMID: 37553253; PMCID: PMC10410066.)

11) donkey anti-Mouse IgG (H+L) Highly Cross-Adsorbed Secondary Antibody, Alexa Fluor 555, (Invitrogen, A31570) Application:Western Blots, Immunohistochemistry (IHC) and others (see https://www.thermofisher.com/antibody/product/Donkey-anti-Mouse-IgG-H-L-Highly-Cross-Adsorbed-Secondary-Antibody-Polyclonal/A-31570) References to the papers which used this antibody can be found at producers webpage (example: Chen J, Neil JA, Tan JP, Rudraraju R, Mohenska M, Sun YBY, Walters E, Bediaga NG, Sun G, Zhou Y, Li Y, Drew D, Pymm P, Tham WH, Rossello FJ, Nie G, Liu X, Subbarao K, Polo JM. A placental model of SARS-CoV-2 infection reveals ACE2-dependent susceptibility and differentiation impairment in syncytiotrophoblasts. Nat Cell Biol. 2023 Aug;25(8):1223-1234. doi: 10.1038/s41556-023-01182-0. Epub 2023 Jul 13. PMID: 37443288; PMCID: PMC10415184.)

Eukaryotic cell lines

Policy information about cell lines and Sex and Gender in Research

Cell line source(s)	Cell lines VeroE6 and MRC5 were obtained from ATCC. HEK293T cells were a gift from Johan Eriksson, Uppsala University, Sweden
Authentication	Cell lines were authenticated by the manufacturers. None of the cell lines were in-house authenticated.
Mycoplasma contamination	Cell line stocks were screened for Mycoplasma and no contamination was detected.
Commonly misidentified lines (See ICLAC register)	No commonly misidentified lines were used.
